# Post-conception heat exposure increases clinically unobserved pregnancy losses

**DOI:** 10.1038/s41598-021-81496-x

**Published:** 2021-01-21

**Authors:** Tamás Hajdu, Gábor Hajdu

**Affiliations:** 1 Institute of Economics, Centre for Economic and Regional Studies, Budapest, Hungary; 2 Institute for Sociology, Centre for Social Sciences, Budapest, Hungary

**Keywords:** Environmental economics, Environmental social sciences

## Abstract

Evidence of the relationship between temperature during pregnancy and human embryo mortality is limited. Most importantly, the literature lacks causal estimations and studies on early pregnancy losses. Here, we estimate the impact of early pregnancy temperature exposure on the clinically unobserved pregnancy loss rate. We use administrative data of clinically observed pregnancies from more than three decades for Hungary. We apply an empirical approach that allows us to infer the impact of temperature on the clinically unobserved pregnancy loss rate from the estimated effects on the clinically observed conception rate. The results show that exposure to hot temperatures during the first few weeks after the conception week increases the clinically unobserved pregnancy loss rate, whereas exposure to colder temperatures seems to decrease it. Importantly, the temperature-induced changes represent changes in the total number of pregnancy losses rather than a compositional change between clinically observed and clinically unobserved pregnancy losses.

## Introduction

The Earth’s climate is rapidly warming. The projected changes have prompted numerous studies on the impact temperature has on natural and human systems^1–3^. A large body of rapidly growing research focuses on the impact of temperature and climate change on human mortality^4–15^. Nevertheless, the evidence concerning the impacts on embryo mortality is still limited. Most previous papers have investigated the relationship between ambient temperature and the risk of late foetal death (stillbirth)^16–22^, while only a few studies have analysed the association of temperature exposure with miscarriage risk^23,24^. One of the main shortcomings of these papers is the lack of causal evidence. Furthermore, due to obvious data limitations, the largest share of pregnancy losses that occur in the early period of pregnancy, namely, clinically unobserved pregnancy losses, are ignored.

Human embryo mortality between fertilization and birth is high. The most reliable estimations range from 40 to 67%^25,26^. Most of these pregnancy losses remain clinically unobserved. The share of conceptions lost before clinical recognition is estimated to be between 20 and 60%^25,26^. A primary cause of this sizeable uncertainty is that embryo mortality from fertilization to implantation is undetectable by any technology. Furthermore, most post-implantation losses occur before the pregnancy becomes clinically recognized. Many early embryo losses are caused by genetic abnormalities^27,28^, although environmental and behavioural factors may also play a role^29,30^. As most pregnancy losses are clinically undetected, we also have to assess clinically unobserved pregnancy losses to completely understand the impact of in utero temperature exposure on human embryo mortality.

Here, we analyse the impact of early pregnancy temperature exposure on the clinically unobserved pregnancy loss rate. Our empirical approach relies on two identification assumptions (for a formal discussion, see Methods). The first assumption is that the total number of conceptions is the sum of conceptions that end in clinically observed pregnancy outcomes and conceptions that end in clinically unobserved pregnancy losses. This assumption holds, at least, in the developed countries. In Hungary, as in other developed countries, administrative registers that cover clinically observed pregnancy outcomes are characterized by completeness. In addition, as access to abortions was not seriously restricted even in the 1980s, illegal abortions (that are likely to be clinically unobserved) are basically non-existent during our sample period^31,32^. Therefore, we can assume that pregnancies ending in a clinically unobserved outcome completely consist of pregnancy losses before clinical recognition. (We note that introducing illegal abortion does not invalidate the analysis and our conclusions. For the details, see Methods.) The second assumption is that the total number of conceptions is not altered by post-conception temperature exposure temperatures. In other words, future weather does not influence how many pregnancies start today. In theory, behavioural changes in response to information on forthcoming weather can occur and may influence the number of conceptions, which would violate this assumption. Although this is very unlikely to be a considerable influencing factor, we directly rule out this possibility with robustness tests. In our sample period, even a 7-day forecast was far from a high degree of accuracy^33^. Building on the limited accuracy of weather forecasts, we show that responses to weather forecasts do not drive the estimated relationship between early pregnancy temperature exposure and the clinically unobserved pregnancy loss rate.

As post-conception temperature exposure is not able to change the total number of conceptions, if we observe that post-conception temperature influences the number of conceptions that end in clinically observed pregnancies, then it means that the number of clinically unobserved pregnancy losses is changed in the opposite direction. In other words, the outcome of some conceptions has changed. That is, even though official statistics do not contain any information on clinically unobserved pregnancy losses, from estimations using data of conceptions ending in clinically observed pregnancies, we can infer the impact of early pregnancy temperature exposure on the clinically unobserved pregnancy loss rate.

We use administrative data from Hungary with full coverage of the clinically observed pregnancies (conceptions) recorded by the country’s health care system. We estimate the impact of early pregnancy temperature exposure (defined as a six-week-long period starting after the week of conception) on the conception rate calculated from clinically observed pregnancies. The outcome variable of our study is the clinically observed conception rate at the county-year-week level. This variable is defined as the number of clinically observed conceptions in a given county per week per 100,000 women aged 16–44 years. Using the information of the last day of pregnancy and pregnancy length, we estimate the date of conception for all pregnancies and identify the year and calendar week of conception. The county of conception is determined by the residence of the mother. Weekly weather data are matched to the conceptions according to the county of the mother’s residence.

To estimate the causal effect of early pregnancy temperature exposure on the observed conception rate, we build on recent studies in the empirical climate economics literature^5–8,34–36^. We exploit the random year-to-year variation in the calendar-week-specific temperature exposure. Our model controls for county-specific shocks at the year level, for differences in region-specific seasonality and its change over time. Intuitively, our model estimates the temperature effects by comparing the clinically observed conception rates of the same calendar week and county across years with warmer and cooler post-conception temperatures after accounting for any county-by-year-specific changes in the observed conception rates and temporal trends in seasonality. We allow for a nonlinear temperature-conception rate relationship by using eight temperature categories that represent the number of days with different daily mean temperatures. The lowest category is ≤  −5 °C and the highest is > 25 °C, and the intermediate categories are 5 °C wide. In the analysis, 15–20 °C serves as the reference category. (Supplementary Table S1 provides summary statistics for our dependent variable and key temperature variables.) We also control for early pregnancy precipitation, pre-conception weather, and the share of non-working days around the conception week.

Our data cover more than 6.5 million pregnancies with conceptions occurring between 1981 and 2015, including live births, miscarriages, stillbirths, and induced abortions, incorporated into 36,400 county-year-week cells. We note again that the impact of the post-conception temperature exposure on the total conception rate must be zero; therefore, the effects on the clinically observed and unobserved conception rates must cancel each other out, which means that the impacts of temperature exposure on the clinically unobserved pregnancy loss rate can be obtained by multiplying the estimated temperature coefficients by − 1. Further details of the data and model can be found in the Methods section.

## Results

We find that early pregnancy temperature exposure to an additional hot day (mean temperature > 25 °C) increases the clinically unobserved pregnancy loss rate by 0.22 pregnancy losses per 100,000 women aged 16–44 years, compared with a day with a mean temperature of 15–20 °C (Table 1). The effects of exposure to days with temperatures of 20–25 °C or 10–15 °C are basically identical to those of exposure to a day with a temperature of 15–20 °C. The coefficients of colder temperature bins are negative, but some of them are not different from zero at the 95% significance level, and the point estimate for the coldest temperature bin is especially close to zero. In general, the impact of temperature exposure during the first 6 weeks of the pregnancy (excluding the conception week) seems to be non-linear. Hot days increase the clinically unobserved pregnancy loss rate, whereas colder days seem to decrease it, but the latter impacts are not particularly different from each other. Our findings are in line with the results of other mammalian studies^37–41^.

**Table 1:** Impact of early pregnancy temperature exposure on the clinically unobserved pregnancy loss rate.

Daily mean temperature (°C)	Coeff	SE	*p* value	95% CI
below –5	–0.036	(0.087)	0.681	[− 0.218; 0.146]
–5 to 0	–0.195	(0.072)	0.013	[− 0.345; − 0.045]
0 to 5	–0.088	(0.062)	0.173	[− 0.217; 0.042]
5 to 10	–0.124	(0.046)	0.015	[− 0.220; − 0.027]
10 to 15	0.016	(0.033)	0.628	[− 0.054; 0.087]
15 to 20	Ref. cat			
20 to 25	0.036	(0.045)	0.437	[− 0.059; 0.131]
over 25	0.225	(0.049)	0.000	[0.122; 0.327]

The coefficients show the impact of early pregnancy temperature exposure by temperature category. The coefficients represent the effect of one additional day with a given mean temperature on the clinically unobserved pregnancy loss rate relative to a day with a mean temperature of 15–20 °C. The early pregnancy period is defined as a six-week-long period starting after the week of conception. The estimations come from Eq. (9). The outcome variable is the clinically observed conception rate per week per 100,000 women aged 16–44 years, which is calculated using conceptions that end in clinically observed pregnancy outcomes. The impacts of temperature exposure on the clinically unobserved pregnancy loss rate are obtained by multiplying the estimated temperature coefficients by − 1. The model has county-by-year fixed effects, region-by-calendar-week fixed effects, and region-by-calendar-week-specific quadratic time trends. Precipitation, pre-conception weather, and the share of non-working days are controlled for. We weight by the counties’ average female population size (aged 16–44 years) between 1981 and 2015. Standard errors are clustered by county and time.

We test the sensitivity of the results by additional model specifications: controlling for lagged values of observed conception rates, applying different functional forms when accounting for changes in county-specific seasonality, using different fixed effects, estimating an unweighted regression (Supplementary Table S2), applying alternative clustering of the standard errors (Supplementary Table S3) and using 3 °C wide temperature categories (Supplementary Table S4). The conclusion remains similar, but the effect of early pregnancy temperature exposure seems to increase at temperatures above 25 °C. In addition, as placebo tests, the temperature and precipitation variables are replaced with weather data that were measured exactly one or two years later. These estimations further support the credibility of the baseline results (Supplementary Table S5).

**Figure 1:**
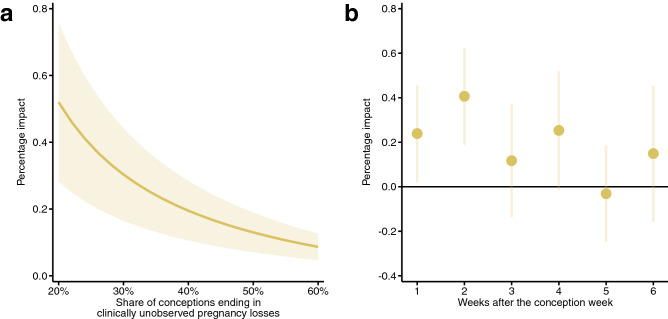
Percentage impact of early pregnancy exposure to a day with a mean temperature above 25 °C on the clinically unobserved pregnancy loss rate. (**a**) The percentage impact of early pregnancy exposure to an additional day with a mean temperature above 25 °C on the clinically unobserved pregnancy loss rate (relative to a day with a mean temperature of 15–20 °C) assuming different values for the share of conceptions that are spontaneously lost before clinical recognition. The early pregnancy period is defined as a six-week-long period starting after the week of conception. (**b**) The percentage impact of exposure to an additional day with a mean temperature above 25 °C on the clinically unobserved pregnancy loss rate by pregnancy week. For this calculation, we assume that 40% of conceptions are lost before clinical recognition. Under this assumption, the baseline weekly rate of clinically unobserved pregnancy losses is 115.2 (per 100,000 women aged 16–44 years). The shaded area and the error bars represent 95% confidence intervals. The estimates come from Eqs. (9) and (13), whereas the percentage impacts are calculated by Eq. (11). The impacts on the clinically unobserved pregnancy loss rate are inferred from regressions with the clinically observed conception rate as the outcome variable. The clinically observed conception rate is calculated using conceptions that end in clinically observed pregnancy outcomes, and it is defined as the number of conceptions per week per 100,000 women aged 16–44 years. The model has county-by-year fixed effects, region-by-calendar-week fixed effects, and region-by-calendar-week-specific quadratic time trends. Precipitation, pre-conception weather, and the share of non-working days are controlled for. We weight by the counties’ average female population size (aged 16–44 years) between 1981 and 2015. Standard errors are clustered by county and time.

**Figure 2:**
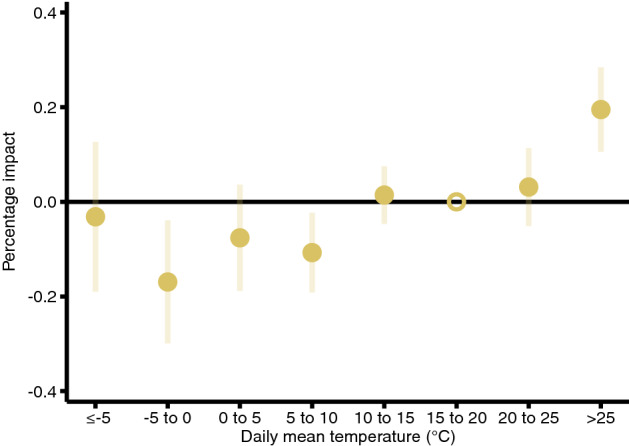
Percentage impact of early pregnancy temperature exposure on the clinically unobserved pregnancy loss rate. The percentage impact of early pregnancy exposure to one additional day with different mean temperatures on the clinically unobserved pregnancy loss rate (relative to a day with a mean temperature of 15–20 °C) assuming that 40% of conceptions are lost before clinical recognition. The early pregnancy period is defined as a six-week-long period starting after the week of conception. The shaded area and the error bars represent 95% confidence intervals. The estimates come from Eq. (9), whereas the percentage impacts are calculated by Eq. (11). See notes to Fig. 1 for details on the estimated model.

Although the estimated coefficients show the impact of temperature exposure on the clinically unobserved pregnancy loss rate (number of clinically unobserved pregnancy losses per week per 100,000 women aged 16–44 years), the calculation of the percentage impact is slightly more challenging. We should simply divide the coefficients by the baseline rate of clinically unobserved pregnancy losses; however, its exact value is unknown. The best available estimations suggest that 20–60% of conceptions are spontaneously lost before clinical recognition^25,26^, which can be used to calculate the baseline weekly rate (see Methods). Under the assumption that 40% of conceptions are lost before clinical recognition, the average clinically unobserved pregnancy loss rate is 115.2 per week per 100,000 women aged 16–44 years, which means that early pregnancy exposure to a day with a mean temperature above 25 °C increases the clinically unobserved pregnancy loss rate by 0.20% (Fig. 1a) relative to exposure to a day in the 15–20 °C temperature bin. Assuming a 20% share, the percentage impact is 0.52%, whereas assuming a 60% share, it is 0.09%. For the colder temperature bins (below 10 °C), under the assumption that 40% of conceptions are lost before clinical recognition, the estimated percentage impacts vary around − 0.10% (Fig. 2).

To rule out that behavioural changes in response to weather forecast influence the number of conceptions and drive our results, we estimate a regression in which the first week of the pregnancy is excluded from the main temperature and precipitation variables. That is, we focus on the period between the second and sixth weeks of the pregnancy. As the accuracy of (long-term) weather forecasts were limited in our sample period^33^, if the temperature coefficients remain generally unchanged in this setting, then we can conclude that response to forthcoming weather is not an important factor in the relationship between early pregnancy temperature exposure and the clinically unobserved pregnancy loss rate. As expected, the results are very similar to the baseline estimation (Supplementary Table S6).

Next, we examine which pregnancy week in the early pregnancy period is the most sensitive to exposure to a hot ambient temperature. In this analysis, we use weekly temperature variables instead of aggregated ones. The results are presented as the percentage impact of temperature exposure on the clinically unobserved pregnancy loss rate, assuming that 40% of conceptions are lost before clinical recognition (Fig. 1b). (Results under different assumptions on the share of unobserved pregnancy losses are shown in Supplementary Fig. S1). We find that exposure to a hot day has the largest impact in the first two weeks after the conception week. The clinically unobserved pregnancy loss rate is increased by 0.24% due to exposure to a hot day in the first week after the conception week and by 0.41% due to exposure to a hot day in the second week. The impacts of exposure to a hot day after the second week start to decrease, approaching zero. This pattern is exactly what we expect, as the likelihood that a pregnancy loss remains clinically unobserved should decrease with the length of the pregnancy. Extending the exposure period up to the eighth week gives similar results (Supplementary Fig. S2). The estimated impacts for weeks 7 and 8 are practically zero, which supports the validity of the interpretation of our findings.

Finally, it is interesting to examine whether exposure to hot weather increases the clinically unobserved pregnancy loss rate because it simply shifts forward the date of some pregnancy losses that would be recorded as clinically observed pregnancy losses without the heat exposure (but becomes clinically unobserved due to exposure to high temperature), or the estimated increase represents a “net” growth in pregnancy losses. To check this hypothesis, we run a regression in which the dependent variable is the conception rate calculated from pregnancies resulting in clinically observed spontaneous foetal losses (miscarriages and stillbirths). As we find temperature coefficients close to zero (Supplementary Table S7), we can conclude that early pregnancy exposure to hot temperature causes an increase in the total number of pregnancies ending in pregnancy loss.

## Discussion

Using administrative data and applying a novel empirical approach, we provide evidence that exposure to hot temperatures during the first few weeks of pregnancy increases the clinically unobserved pregnancy loss rate. This increase in the clinically unobserved pregnancy loss rate means a “net” increase in total pregnancy losses rather than a compositional change between clinically observed and clinically unobserved pregnancy losses. In other words, early pregnancy exposure to hot temperatures decreases the chance that a pregnancy ends in a clinically observed outcome (live birth, induced abortion, miscarriage/stillbirth) and increases the chance of an early (clinically unobserved) embryo loss.

We find that each additional > 25 °C day during the early pregnancy period causes an increase of 0.22 pregnancy losses in the clinically unobserved pregnancy loss rate, which reflects an increase of 0.09% to 0.52%, depending on the assumption regarding the share of conceptions that are lost before clinical recognition. The impacts are especially high during the first few weeks after the conception week, whereas they become practically zero after the sixth week, which is in line with the fact that the longer the pregnancy is, the higher the likelihood of clinical recognition.

Most previous papers have examined the association between ambient temperature and stillbirths, which is typically defined as foetal death after the 20th/28th pregnancy week and constitutes a very small fraction of pregnancy losses. They analysed the impacts during different periods of pregnancy. Therefore, a direct comparison of our estimations with these studies is difficult. While previous studies have found that temperature during the later periods of the pregnancy is associated with late foetal losses, our study provides the first estimate of a causal relationship between early pregnancy temperature exposure and the clinically unobserved pregnancy loss rate that covers the majority of pregnancy losses.

This paper provides important implications for the wider literature as well. Our estimations provide evidence that early pregnancy temperature exposure changes the composition of foetuses that survive to live birth (“culling” effect). Therefore, the subsequent birth cohort represents a selected sample of conceptions. The selection is unlikely to be random, but it is likely to remove foetuses with below-average health^42–44^. Several papers have aimed to estimate the “scarring” effect of in-utero temperature exposure on health at birth and found that exposure to heat reduces birth weight. Our results imply that these estimations are very likely to be biased towards the opposite direction (biased upwards) due to selection in utero. Estimations of the impacts of temperature exposure during early pregnancy should be especially affected. Indeed, the most reliable estimates show that the effect of first trimester exposure to a hot temperature on birth weight is much weaker than the effect of exposure to a hot temperature during the second and third trimesters^45–47^, which is consistent with a selection-induced upward bias, although other factors may also play a role. More importantly, our empirical approach offers a tool that can be used for the estimation of a corrected scarring effect. Calculating the conception rate based on pregnancies ending in live births and regressing it on the post-conception exposure yields an estimation of the extent of the culling effect. With a further assumption of the average difference between the birth weight of the observed new-borns and the birth weight of the “culled” foetuses, a bias-corrected estimation of the scarring effect can be obtained.

Some limitations of this study should be mentioned. First, the data we used do not allow us to identify and understand the mechanisms through which temperature influences early pregnancy losses. Second, the accuracy of the conceptions rates might be affected by residential mobility during pregnancy and the estimation of the date of conception, albeit the influence of these factors is likely to be limited. From a European perspective, residential mobility in Hungary is low^48^, and a large fraction of residential migrations occurs over a short distance^49^, therefore, for most women, the county of residence at the end of pregnancy is identical to the county of residence during pregnancy. Although the estimated date of conception is very likely to be biased for many pregnancies, this bias is unlikely to seriously affect the conception rates. For most pregnancies, a small bias (few days) does not change the (estimated) week of conception. In addition, the number of conceptions that is added to and subtracted from a given week due to the inaccuracy of the conception dates are likely to be roughly equal, therefore the estimated and actual conception rates should not be too different from each other. Furthermore, if the bias is statistically independent of the explanatory variables, our estimations are still unbiased, but the precision is reduced^50^. Finally, we note that our results are based on data from Hungary and cannot necessarily be generalized to other countries. In addition, future adaptation could mitigate the impacts of temperature exposure on early pregnancy losses. More research is needed to analyse the role of adaptation and how early pregnancy temperature exposure affect clinically unobserved pregnancy losses in other parts of the world.

## Methods

### Clinically observed conceptions

Clinically observed conceptions are conceptions that end in clinically observed pregnancy outcomes, whereas clinically unobserved conceptions are conceptions that end in clinically unobserved pregnancy losses. To calculate clinically observed conception rates, we use the administrative registers of the Hungarian Central Statistical Office. These registry data cover all clinically observed pregnancies (conceptions) that ended in a live birth, miscarriage, stillbirth or induced abortions between 1981 and 2016 in Hungary. We accessed the de-identified datasets in the secure data environment of the HCSO after an accreditation process.

We estimate the date of conception for all pregnancies using information of the date of birth/abortion/pregnancy loss (LD) and gestation length (GL). First, we estimate the first day of the last menses. In the administrative data we use, gestation length (for all type of pregnancies) is calculated from the first day of the last menses, which is self-reported by the woman, however, is excluded from the datasets. Gestation length is available in completed weeks. We estimate the beginning of the menstrual cycle as follows:

1 $${\text{M}} = {\text{LD}} - \left( {{\text{GL}} \times 7 + 3} \right)$$

where M is the first day of the last menses, LD is the last day of the pregnancy, and GL is gestation length (reported in completed weeks). Because GL is recorded in completed weeks, the actual gestational age is 0–6 days longer than the reported one. Therefore, we calculate M by adding 3 days to the reported pregnancy length (GL).

In the second step, we estimate the date of conception based on M. As conception (fertilization) occurs within hours after ovulation^51,52^ and the day of ovulation is most likely to be between the 11th and 19th day of the menstrual cycle^51,53–56^, we assume that conceptions occur on the 15th day:

2 $${\text{FD}} = {\text{M}} + 14$$

where FD is the first day of the pregnancy (conception day), and M is the first day of the last menses.

Based on the estimated conception dates, we calculate clinically observed conception rates at the county-year-week level defined as the number of clinically observed conceptions per week per 100,000 women aged 16–44 years. We divide each year into 52 weeks, which means that the last week is 8 days long (except in leap years when it lasts 9 days). The county of conception (pregnancy) is defined by the place of residence of the mother (at the end of pregnancy). Budapest, the capital of Hungary, is a separate administrative unit; therefore, it is considered to be an individual county (in accordance with the NUTS, Nomenclature of Territorial Units for Statistics, classification system). The number of women aged 16–44 years (at the beginning of the year) for every county comes from the Hungarian Central Statistical Office. These population figures are assigned to the first week of the year, and the unobserved county-week figures are estimated by linear interpolation between the years.

Pregnancies with missing information on gestational age or on the exact day of the end of the pregnancy are excluded, as well as pregnancies with non-Hungarian or unknown places of residence (less than 1% of all clinically observed pregnancies in total). Our final sample covers 6,544,519 clinically observed pregnancies (3,722,068 live births, 2,228,219 induced abortions, and 594,232 spontaneous foetal deaths) with conception days estimated to be between 1981 and 2015.

Finally, we note that the estimated conception date of a given pregnancy might be a biased estimation of the actual conception date, but the bias affects our conception rates if, and only if, it changes the (estimated) week of the conceptions. As we calculate conception rates at the week level, for most pregnancies, a small bias (a couple of days) in the estimated conception date does not change the (estimated) week of conception In addition, although some conceptions might be incorrectly assigned to given conception week (lets label it conception week “A”)—falsely increasing the number of conceptions in conception week “A” –, others might be incorrectly assigned to the previous or next weeks (falsely decreasing the number of conceptions in conception week “A”). The net impact of these biases on the conception rates is much lower than the total number of the biased categorizations would suggest. Importantly, if the bias is random, the estimated impacts of temperature exposure are unbiased, albeit they are measured with less accuracy^50^.

### Weather data

We use weather data from the E–OBS 20.0e dataset of the European Climate Assessment & Dataset project^57^, which provides daily weather measures for Europe with a spacing of 0.1° × 0.1° in regular latitude/longitude coordinates from 1950 to 2019. The dataset includes information on the maximum, minimum and mean temperatures and precipitation. We create eight binary temperature variables based on the mean temperature (below − 5 °C, − 5 to 0 °C, 0 to 5 °C, 5 to 10 °C, 10 to 5 °C, 15 to 20 °C, 20 to 25 °C, over 25 °C) and five precipitation variables indicating the amount of daily precipitation (0 mm, 0–3 mm, 3–5 mm, 5–10 mm, over 10 mm) to describe the daily weather conditions at the grid points within Hungary. Next, to preserve the variation in temperature, we average the new temperature and precipitation variables for each day over grid points within the twenty counties of Hungary (including Budapest).

Finally, we construct weekly level measures from the daily data by summing the variables over the weeks for each county. Accordingly, eight temperature variables show the number of days in a given week and given county when the daily mean temperature falls in a certain temperature bin (below − 5 °C, − 5 to 0 °C, 0 to 5 °C, 5 to 10 °C, 10 to 15 °C, 15 to 20 °C, 20 to 25 °C, over 25 °C), and five precipitation variables show the number of days when the amount of daily precipitation falls in a certain precipitation bin (0 mm, 0–3 mm, 3–5 mm, 5–10 mm, over 10 mm).

The weather data are matched to the conceptions by the county of the mother’s residence (at the end of pregnancy).

### Identification assumptions

Our analysis relies on two identification assumptions. First, the total number of conceptions (C) occurring at time *t* is the sum of conceptions that end in clinically observed pregnancy outcomes (C^O^) and conceptions that end in clinically unobserved pregnancy losses (C^L^):

3 $${\text{C}}_{{\text{t}}} = {\text{C}}_{{\text{t}}}^{{\text{O}}} + {\text{C}}_{{\text{t}}}^{{\text{L}}}$$

The second assumption is that the total number of conceptions are not altered by temperatures in the post-conception period. In other words, future weather does not influence how many pregnancies start today:

4 $$\left( {{\text{C}}_{{\text{t}}} |{\text{T}}_{{\text{t + i}}} = 1} \right) - \left( {{\text{C}}_{{\text{t}}} |{\text{T}}_{{\text{t + i}}} = 0} \right) = 0$$

where T is an indicator function that shows, e.g. the occurrence of unusually cold/hot temperature at time *t* + *i*, *i* = 1, 2, …, ∞.

Although, under this assumption, the post-conception temperature is not able to change the total number of conceptions that have already occurred, it can change the outcome of some pregnancies. Thus, post-conception temperature exposure can “influence” both the (a posteriori estimated) number of conceptions that end in clinically observed pregnancy outcomes and the (a posteriori estimated) number of conceptions that end in clinically unobserved pregnancy losses. However, these changes have to cancel each other out so that the total impact equals to zero. Substituting the right-hand side of Eq. (3) into Eq. (4) and rearranging, we have:

5 $$\left[ {\left( {{\text{C}}_{{\text{t}}}^{{\text{O}}} |{\text{T}}_{{\text{t + i}}} = 1} \right) - \left( {{\text{C}}_{{\text{t}}}^{{\text{O}}} |{\text{T}}_{{\text{t + i}}} = 0} \right)} \right] + \left[ {\left( {{\text{C}}_{{\text{t}}}^{{\text{L}}} |{\text{T}}_{{\text{t + i}}} = 1} \right) - \left( {{\text{C}}_{{\text{t}}}^{{\text{L}}} |{\text{T}}_{{\text{t + i}}} = 0} \right)} \right] = 0$$

It means that although the impact of early pregnancy temperature exposure on clinically unobserved pregnancy losses cannot be directly estimated, it can be obtained by multiplying the estimated impact on clinically observed conceptions by − 1. To see this, we can rearrange Eq. (5) as follows:

6 $$\left[ {\left( {{\text{C}}_{{\text{t}}}^{{\text{L}}} |{\text{T}}_{{\text{t + i}}} = 1} \right) - \left( {{\text{C}}_{{\text{t}}}^{{\text{L}}} |{\text{T}}_{{\text{t + i}}} = 0} \right)} \right] = - 1 \times \left[ {\left( {{\text{C}}_{{\text{t}}}^{{\text{O}}} |{\text{T}}_{{\text{t + i}}} = 1} \right) - \left( {{\text{C}}_{{\text{t}}}^{{\text{O}}} |{\text{T}}_{{\text{t + i}}} = 0} \right)} \right]$$

The term in the square bracket in the right-hand side of Eq. (6) shows how post-conception temperature exposure influences the (a posteriori estimated) number of conceptions that end in clinically observed pregnancy outcomes. It can be estimated and empirically analysed. Although the left-hand side of Eq. (6) cannot be directly observed, it can be derived from the impacts on the conceptions that end in clinically observed pregnancy outcomes.

It is important to note that both identification assumptions can be questioned. First, most illegal abortions are clinically unobserved, and therefore they are not covered by the administrative datasets. Thus, if the incidence of illegal abortion is not zero, Eq. (3) does not hold. However, in Hungary, administrative registers that cover clinically observed pregnancy outcomes (live births, induced abortions, miscarriages, and stillbirths) are characterized by completeness. In addition, as access to abortions was not seriously restricted even in the 1980s, illegal abortion is practically negligible, non-existent^31,32^. Therefore, it is reasonable to assume that pregnancies ending in a clinically unobserved outcome completely consist of pregnancy losses before clinical recognition. Importantly, introducing illegal abortion as another type of clinically unobserved conceptions does not invalidate our analysis. In this case, the total number of conceptions is the sum of conceptions that end in clinically observed pregnancy outcomes, conceptions that end in clinically unobserved pregnancy losses and conceptions that end in illegal abortions. Accordingly, Eq. (3) can be rewritten as:

7 $${\text{C}}_{{\text{t}}} = {\text{C}}_{{\text{t}}}^{{\text{O}}} + {\text{C}}_{{\text{t}}}^{{\text{L}}} + {\text{C}}_{{\text{t}}}^{{\text{A}}}$$

where C is the total number of conceptions, C^O^ is the number of conceptions that end in clinically observed pregnancy outcomes, C^L^ is the number of conceptions that end in clinically unobserved pregnancy losses, whereas C^A^ is the number of conceptions that end in (clinically unobserved) illegal abortions. Note that Eq. (6) still holds if the number of conceptions that end in illegal abortions is unaffected by post-conception temperature exposure. In other words, post-conception temperature exposure does not influence the share of clinically unobserved abortions, which is not a very strong assumption:

8 $$\left( {{\text{C}}_{{\text{t}}}^{{\text{A}}} |{\text{T}}_{{\text{t + i}}} = 1} \right) - \left( {{\text{C}}_{{\text{t}}}^{{\text{A}}} |{\text{T}}_{{\text{t + i}}} = 0} \right) = 0$$

If Eq. (8) holds, plugging Eq. (7) into Eq. (4) gives back to Eq. (5). Therefore, from an estimation using data of clinically observed conceptions, we can infer the impact of early pregnancy temperature exposure on the clinically unobserved pregnancy loss rate. Finally, we note that if Eq. (8) would be violated and a significant part of the estimated temperature-induced changes in the number of clinically observed conceptions (see Table 1) is due to changes in the number of (clinically unobserved) illegal abortions, then either the number of illegal abortions should be enormously high (compared to the number of legal abortions) or the post-conception temperature impact on conceptions ending in illegal abortions should be extremely powerful (in a percentage term). Both are basically impossible and contradicts the known facts^31,32^. Accordingly, even if Eq. (8) is violated, the temperature-induced changes in the number of illegal abortions should be close to zero. Therefore, our indirect approach of estimating the impact of early pregnancy temperature exposure on the clinically unobserved pregnancy loss rate is still basically valid.

Regarding the second assumption, one can argue that behavioural changes in response to information on forthcoming temperatures may influence the number of conceptions, which could violate Eq. (4). In this case, the impacts of early pregnancy temperature exposure on the clinically unobserved pregnancy loss rate cannot be inferred from the estimations using data of clinically observed pregnancies. However, in our sample period, even a 7-day forecast was far from a high degree of accuracy, albeit weather forecasts have improved rapidly in the last few decades^33^. Building on the limited accuracy of (long-term) weather forecasts, we can test whether responses to information on forthcoming weather could drive the estimated relationship between early pregnancy temperature exposure and the clinically unobserved pregnancy loss rate or not. For the details of this analysis, see the Empirical methods section.

### Empirical methods

We model the relationship between early pregnancy temperature and the clinically observed conception rate at the county-week level. We estimate the following equation via ordinary least squares:

9 $${\text{Y}}_{{{\text{ct}}}} = \sum\limits_{{\text{j}}} {\upbeta ^{{\text{j}}} } \sum\limits_{{{\text{i}} = 1}}^{6} {{\text{T}}_{{\text{c(t + i)}}}^{{\text{j}}} } + \sum\limits_{{\text{k}}} {\upgamma ^{{\text{k}}} } \sum\limits_{{\text{i = 1}}}^{6} {{\text{P}}_{{\text{c(t + i)}}}^{{\text{k}}} } +\uptau {\text{X}}_{{\text{t}}} +\upomega _{{{\text{rw}}}} +\upeta _{{{\text{cy}}}} +\uplambda _{{{\text{rw}}}} \times \left( {{\text{t + t}}^{2} } \right) +\upvarepsilon _{{{\text{ct}}}}$$

where Y is the clinically observed conception rate in county *c* at time *t* (year *y*, calendar week *w*). T is a vector of weekly level temperature variables indicating the number of days when the daily mean temperatures are: below − 5 °C, − 5 to 0 °C, 0 to 5 °C, 5 to 10 °C, 10 to 15 °C, 15 to 20 °C, 20 to 25 °C, or above 25 °C (these categories are indicated by *j*). In the analysis, the temperature bin of 15–20 °C is the omitted category. For the baseline estimation, we use temperature variables that show the distribution of the daily mean temperature during the first six weeks of the pregnancy (excluding the week of conception) for conceptions started at time *t* in county *c*. Namely, the temperature variables entered in the regression are the total exposures from week *t* + 1 to week *t* + 6. P is a vector of precipitation controls that shows the number of days when the amount of daily precipitation falls in precipitation bin *k* (0–3 mm, 3–5 mm, 5–10 mm, over 10 mm). The omitted category is the number of days without precipitation. X is a vector of control variables and includes the lagged weekly level temperature and precipitation variables, as observed conception rates could be affected by pre-conception weather via changes in sexual behaviour or reproductive health^58–65^ and post-conception weather might be correlated with pre-conception weather. We control for weather in the week of conception and the previous five weeks (separately for each week). Importantly, our large sample sizes allow us to control the independent effects of pre- and post-conception temperatures. X also includes the share of weekend days and holidays that fall on weekdays in the conception week and in the previous week, as they can help to improve the precision of the estimation. Although these weekend days and holidays are uncorrelated with temperature, sexual activity is reported to be driven by holidays and cultural/religious celebrations^66^.

County-by-year fixed effects (η_cy_) control for county-specific shocks in a given year. Region-by-calendar-week fixed effects (ω_rw_) account for seasonal differences in observed conception rates across larger regions of Hungary. We also allow region-specific seasonality to change over time by adding region-by-calendar-week-specific quadratic time trends (λ_rw_). In summary, the effect of temperature on the conception rates calculated from clinically observed pregnancies is identified from the inter-annual variation in the calendar-week-specific temperature exposure after adjusting for differences in region-specific seasonality and its change over time, as well as for county-specific shocks to conception rate at the year level. Regressions are weighted by the counties’ average female population size (aged 16–44) between 1981 and 2015. Standard errors are clustered by county and time (two-way clustering).

The impact of temperature exposure is captured by the β coefficient in Eq. (9), which shows the effect of one additional day when the daily mean temperature falls into temperature bin *j* on the observed conception rate (relative to a day with a mean temperature of 15–20 °C). As noted earlier, temperature exposure during the early stage of pregnancy cannot influence the total conception rate (the sum of the observed and the unobserved conception rates); therefore, − β shows the impact of temperature exposure on the clinically unobserved pregnancy loss rate. Although in this way we obtain an estimation of the impact on the clinically unobserved pregnancy loss rate (number of clinically unobserved pregnancy loss per week per 100,000 women aged 16–44 years), the main difficulty is the calculation of the percentage impact. We should simply divide the − β coefficients by the baseline clinically unobserved pregnancy loss rate; however, the baseline rate is unknown. The best available estimations suggest that 20–60% of conceptions are spontaneously lost before clinical recognition^25,26^. We can use these estimations to calculate a baseline weekly rate of clinically unobserved pregnancy losses:

10 $${\text{FLR}}^{{\text{u}}} = {\text{s}} \times \frac{{{\text{CR}}^{{\text{o}}} }}{{\left( {1 - {\text{s}}} \right)}}$$

where FLR^u^ is the clinically unobserved pregnancy loss rate, CR^o^ is the clinically observed conception rate (the average rate in our sample; 172.75), and *s* is the share of conceptions that are spontaneously lost before clinical recognition. We choose 40% as the default value of *s*, but we provide our results over the whole 20–60% range. Next, Eq. (10) can be used for the calculation of percentage impact of temperature exposure on clinically unobserved pregnancy loss rate:

11 $${\text{b}} = \frac{{ -\upbeta }}{{{\text{FLR}}^{{\text{u}}} }} = \frac{{ -\upbeta }}{{{\text{s}} \times \frac{{{\text{CR}}^{{\text{o}}} }}{{\left( {1 - {\text{s}}} \right)}}}}$$

where b is the estimated percentage impact of temperature exposure on the clinically unobserved pregnancy loss rate under the assumption that the share of conceptions that are spontaneously lost before clinical recognition is *s*.

We note that the effect of early pregnancy temperature exposure on clinically unobserved pregnancy losses might be slightly underestimated by our empirical approach because the impacts of the very first few days in the week of conception are excluded. We do not consider the week of conception as a part of the early pregnancy period because we want to rule out that any impacts of temperature exposure on changes in pre-conception factors (e.g., sexual behaviour or reproductive health) influence our estimates.

As discussed above, behavioural changes in response to weather forecast may influence the number of conceptions, which would be a violation of our second identification assumption. Building on the limited accuracy of (long-term) weather forecasts^33^, we can test this possibility. We rewrite Eq. (9) and exclude temperature in week *t* + 1 from the main right-hand side variables. Thus, they measure the total exposures from week *t* + 2 to week *t* + 6. We note that weather variables of week *t* + 1 are controlled for, as they can be correlated with weathers in the later weeks. If behavioural changes due to information from weather forecasts drive the relationship between early pregnancy temperature exposure and the clinically unobserved pregnancy loss rate, then the β coefficients should be substantially different from those estimated in Eq. (9). Formally, we estimate the following equation:

12 $$\begin{aligned} {\text{Y}}_{{{\text{ct}}}} & = \sum\limits_{{\text{j}}} {\upbeta ^{{\text{j}}} } \sum\limits_{{{\text{i}} = 2}}^{6} {{\text{T}}_{{\text{c(t + i)}}}^{{\text{j}}} } + \sum\limits_{{\text{k}}} {\upgamma ^{{\text{k}}} } \sum\limits_{{\text{i = 2}}}^{6} {{\text{P}}_{{\text{c(t + i)}}}^{{\text{k}}} } + \sum\limits_{{\text{j}}} {\upphi ^{{\text{j}}} {\text{T}}_{{\text{c(t + 1)}}}^{{\text{j}}} } \sum\limits_{{\text{k}}} {\updelta ^{{\text{k}}} {\text{P}}_{{\text{c(t + 1)}}}^{{\text{k}}} } \\ & \quad { + \uptau }{\text{X}}_{{\text{t}}} +\upomega _{{{\text{rw}}}} +\upeta _{{{\text{cy}}}} +\uplambda _{{{\text{rw}}}} \times \left( {{\text{t + t}}^{2} } \right) +\upvarepsilon _{{{\text{ct}}}} \\ \end{aligned}$$

We also examine which pregnancy week is the most sensitive to temperature exposure. In this exercise, instead of the aggregated weather variables, we use the weekly values:

13 $${\text{Y}}_{{{\text{ct}}}} = \sum\limits_{{\text{j}}} {\sum\limits_{{{\text{i}} = 1}}^{6} {\upbeta _{{\text{i}}}^{{\text{j}}} {\text{T}}_{{\text{c(t + i)}}}^{{\text{j}}} } } + \sum\limits_{{\text{k}}} {\sum\limits_{{\text{i = 1}}}^{6} {\upgamma _{{\text{i}}}^{{\text{j}}} {\text{P}}_{{\text{c(t + i)}}}^{{\text{k}}} } } +\uptau {\text{X}}_{{\text{t}}} +\upomega _{{{\text{rw}}}} +\upeta _{{{\text{cy}}}} +\uplambda _{{{\text{rw}}}} \times \left( {{\text{t + t}}^{2} } \right) +\upvarepsilon _{{{\text{ct}}}}$$

We note that—like Eq. (12)–Eq. (13) also helps to rule out the potential bias arising from the influence of weather forecasts. In that estimation, insignificant and close to zero temperature coefficients after the second week may generate concerns about our interpretations of the estimations. In contrast, significant coefficients can rule out the influence of weather forecasts.

To test the sensitivity of the baseline results, we estimate additional model specifications. We include lagged values of the clinically observed conception rate (up to 10 weeks). We estimate our model by including region-by-week-specific linear or cubic time trends instead of the baseline region-by-week-specific quadratic time trends, changing the fixed effects, and applying alternative clustering of the standard errors. We also estimate an unweighted regression, and a specification using 3 °C wide temperature categories (≤ –6 °C, − 6 to − 3 °C, …, 24 to 27 °C, > 27 °C).

Furthermore, as placebo checks, the temperature and precipitation variables in the baseline model are replaced with weather data that were measured exactly one or two years later. Because clinically unobserved pregnancy loss rates could not have been affected by temperature in the distant future, zero or close to zero coefficients should be observed in the placebo regressions if our identification strategy is valid.

### Ethical approval

No ethical approval and consent from participants were needed for this study because it is based on secondary analysis of the administrative data obtained from the Hungarian Central Statistical Office (HCSO). The study uses completely anonymized administrative data (vital statistics: live birth, induced abortion, and foetal loss registers) with no identifiable information. We accessed the de-identified datasets in the secure data environment of the HCSO after an accreditation process. All researchers were required to sign a contract and a confidentiality commitment.

## Supplementary Information

Supplementary Information.

## Data Availability

All data and code necessary for replication of the results in this paper are available for download at https://figshare.com/s/c8c27b1b54cd0acebe1d. The original weather data can be downloaded from: https://www.ecad.eu//download/ensembles/download.php. Clinically observed conception data has been produced using the live birth, induced abortion, and foetal loss registers of the Hungarian Central Statistical Office (HCSO). The de-identified microdata sets are available only for research purposes in a secure data environment (HCSO–CERS research room).
