# Supplementary material for: Post-conception heat exposure increases clinically unobserved pregnancy losses
Source: Sci Rep. 2021 Jan 21;11:1987. doi: 10.1038/s41598-021-81496-x (PMC7820015; doi:10.1038/s41598-021-81496-x)
Supplement: Supplementary file 1 — Supplementary Information. [file 41598_2021_81496_MOESM1_ESM.pdf]

Supplementary Materials for

Post-conception heat exposure increases clinically unobserved pregnancy losses

Tamás Hajdu[a,*] and Gábor Hajdu[b]

[a] Institute of Economics, Centre for Economic and Regional Studies, Hungary (hajdu.tamas@krtk.mta.hu)
[b] Institute for Sociology, Centre for Social Sciences, Hungary (hajdu.gabor@tk.mta.hu)
[*] corresponding author

This PDF file includes:

Figure S1-S2

Table S1-S7

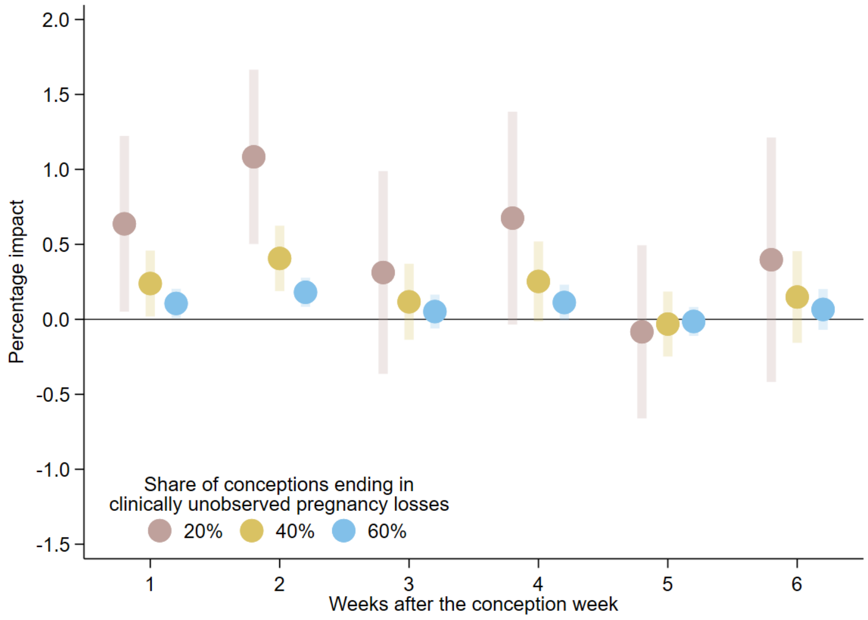

Supplementary Figure S1: Percentage impact of early pregnancy exposure to hot temperature on the clinically unobserved pregnancy loss rate by pregnancy week

The percentage impact of exposure to an additional day with a mean temperature above 25°C on the clinically unobserved pregnancy loss rate by pregnancy week. For this calculation, we assume that 20%, 40%, or 60% of conceptions are lost before clinical recognition. The error bars represent 95% confidence intervals. The estimates come from the same model as Fig. 1b.

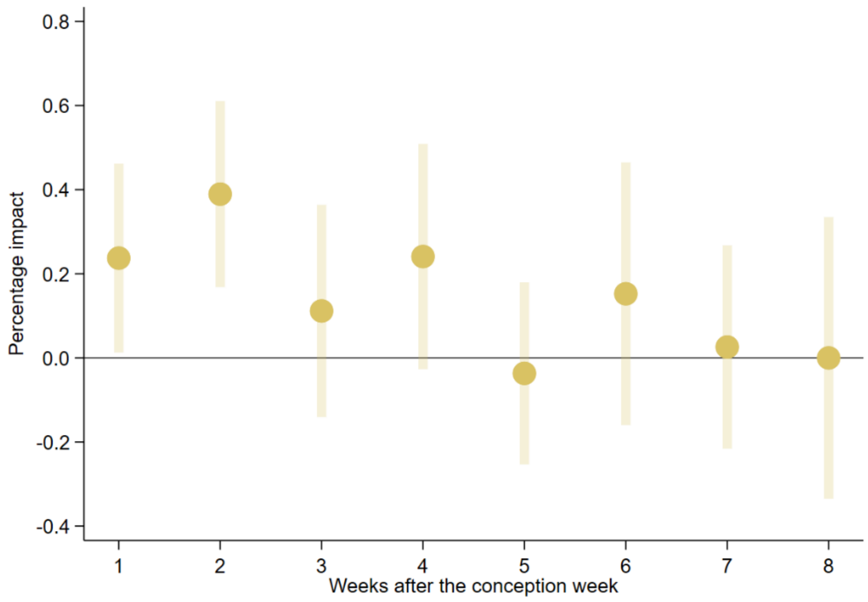

Supplementary Figure S2: Impact of early pregnancy exposure to hot temperature on the clinically unobserved pregnancy loss rate by pregnancy week

The percentage impact of exposure to an additional day with a mean temperature above 25°C on the clinically unobserved pregnancy loss rate by pregnancy week. For this calculation, we assume that 40% of conceptions are lost before clinical recognition. The error bars represent 95% confidence intervals. The estimates come from the same model as Fig. 1b, except we include temperature (and precipitation) exposures in the seventh and eighth weeks after the conception week.

Supplementary Table S1: Descriptive statistics

	mean	SD	min	max	N
Clinically observed conception rate	172.8	34.9	66.9	349.6	36,400
N of days during the early pregnancy period with a mean temperature					
≤−5°C	1.3	3.4	0.0	28.3	36,400
−5 to 0°C	4.1	6.6	0.0	33.5	36,400
0 to 5°C	6.9	8.3	0.0	33.6	36,400
5 to 10°C	6.7	7.3	0.0	33.3	36,400
10 to 15°C	7.1	7.7	0.0	38.8	36,400
15 to 20°C	8.7	9.1	0.0	38.5	36,400
20 to 25°C	6.1	8.8	0.0	38.7	36,400
>25°C	1.3	3.3	0.0	26.3	36,400

Notes: Units of observations: county-by-year-by-week. Weighted by the average female population (aged 16–44) of the counties between 1982 and 2015. The clinically observed conception rate (per week per 100,000 women aged 16–44) is defined as the number of conceptions that end in clinically observed pregnancy outcomes. Each year is divided into 52 weeks, therefore calendar week 52 is 8 days long (except leap years, when it lasts 9 days). The early pregnancy period is defined as a six-week-long period starting after the week of conception.

Supplementary Table S2: Sensitivity of the estimates of early pregnancy temperature exposure on the clinically unobserved pregnancy loss rate

Daily mean temperature (°C)	(1)	(2)	(3)	(4)	(5)	(6)
below –5	–0.032 (0.083)	–0.025 (0.090)	–0.054 (0.087)	–0.030 (0.085)	–0.025 (0.089)	–0.011 (0.091)
–5 to 0	–0.179* (0.068)	–0.164* (0.073)	–0.189* (0.073)	–0.150^{+} (0.072)	–0.193* (0.074)	–0.192* (0.071)
0 to 5	–0.075 (0.057)	–0.095 (0.059)	–0.083 (0.063)	–0.047 (0.057)	–0.084 (0.063)	–0.071 (0.062)
5 to 10	–0.112* (0.043)	–0.115* (0.045)	–0.133** (0.046)	–0.082 (0.054)	–0.120* (0.048)	–0.126* (0.050)
10 to 15	0.022 (0.030)	0.015 (0.032)	0.024 (0.033)	0.003 (0.031)	0.015 (0.035)	0.028 (0.033)
15 to 20	ref. cat.	ref. cat.	ref. cat.	ref. cat.	ref. cat.	ref. cat.
20 to 25	0.032 (0.041)	0.026 (0.043)	0.036 (0.045)	0.038 (0.044)	0.038 (0.046)	0.025 (0.042)
over 25	0.213** (0.045)	0.226** (0.049)	0.222** (0.050)	0.291** (0.049)	0.228** (0.052)	0.202** (0.062)
Fixed effects 1.	County-year	County-year	County-year	County	County-year	County-year
Fixed effects 2.	Region-calendar week	Region-calendar week	Region-calendar week	Year	County-calendar week	Region-calendar week
Fixed effects 3.				Calendar week		
Time trends	Region-by-calendar-week-specific quadratic	Region-by-calendar-week-specific linear	Region-by-calendar-week-specific cubic		County-by-calendar-week-specific quadratic	Region-by-calendar-week-specific quadratic
Lagged conception rate	Yes	No	No	No	No	No
Weighted	Yes	Yes	Yes	Yes	Yes	No

The coefficients show the impact of early pregnancy temperature exposure by temperature category. The coefficients represent the effect of one additional day with a given mean temperature on the clinically unobserved pregnancy loss rate relative to a day with a mean temperature of 15–20°C. The early pregnancy period is defined as a six-week-long period starting after the week of conception. The outcome variable is the clinically observed conception rate per week per 100,000 women aged 16–44 years, which is calculated using conceptions that end in clinically observed pregnancy outcomes. The impacts of temperature exposure on the clinically unobserved pregnancy loss rate are obtained by multiplying the estimated temperature coefficients by −1. The models include different fixed effects as indicated in the “Fixed effects” rows. Some models also contain time trends, lagged conception rates, and are weighted by the counties’ average female population size (aged 16–44 years) between 1981 and 2015, as indicated in the bottom three rows. Precipitation, pre-conception weather, and the share of non-working days are controlled for. Standard errors are shown in parenthesis, clustered by county and time. $^{+}p<0.10$, $^{*}p<0.05$, $^{**}p<0.01$

Supplementary Table S3: Estimates of early pregnancy temperature exposure on the clinically unobserved pregnancy loss rate applying different ways of clustering the standard errors

Daily mean temperature (°C)	(1)	(2)	(3)	(4)	(5)
below –5	–0.036 (0.087)	–0.036 (0.038)	–0.036 (0.094)	–0.036 (0.046)	–0.036 (0.106)
–5 to 0	–0.195* (0.072)	–0.195** (0.048)	–0.195** (0.067)	–0.195** (0.058)	–0.195 (0.113)
0 to 5	–0.088 (0.062)	–0.088^{+} (0.049)	–0.088 (0.055)	–0.088 (0.055)	–0.088 (0.088)
5 to 10	–0.124* (0.046)	–0.124** (0.031)	–0.124* (0.051)	–0.124** (0.040)	–0.124 (0.088)
10 to 15	0.016 (0.033)	0.016 (0.026)	0.016 (0.033)	0.016 (0.031)	0.016 (0.059)
15 to 20	ref. cat.	ref. cat.	ref. cat.	ref. cat.	ref. cat.
20 to 25	0.036 (0.045)	0.036 (0.039)	0.036 (0.036)	0.036 (0.043)	0.036 (0.062)
over 25	0.225** (0.049)	0.225** (0.035)	0.225** (0.053)	0.225** (0.045)	0.225* (0.083)
Clustering	County + Time	County	Time	County + Region-Year	County + Year

The coefficients show the impact of early pregnancy temperature exposure by temperature category. The coefficients represent the effect of one additional day with a given mean temperature on the clinically unobserved pregnancy loss rate relative to a day with a mean temperature of 15–20°C. The early pregnancy period is defined as a six-week-long period starting after the week of conception. The estimations come from equation (9). The outcome variable is the clinically observed conception rate per week per 100,000 women aged 16–44 years, which is calculated using conceptions that end in clinically observed pregnancy outcomes. The impacts of temperature exposure on the clinically unobserved pregnancy loss rate are obtained by multiplying the estimated temperature coefficients by −1. The model has county-by-year fixed effects, region-by-calendar-week fixed effects, and region-by-calendar-week-specific quadratic time trends. Precipitation, pre-conception weather, and the share of non-working days are controlled for. We weight by the counties' average female population size (aged 16–44 years) between 1981 and 2015. Columns show estimates applying different clustering schemes as indicated in the bottom row. Standard errors are shown in parenthesis. $^{+}p<0.10$, $^{*}p<0.05$, $^{**}p<0.01$

Supplementary Table S4: Impact of early pregnancy temperature exposure on the clinically unobserved pregnancy loss rate using 3°C-wide temperature categories

Daily mean temperature (°C)	(1)
below –6	–0.014 (0.107)
–6 to –3	–0.098 (0.083)
–3 to 0	–0.184^{+} (0.095)
0 to 3	–0.026 (0.064)
3 to 6	–0.087 (0.079)
6 to 9	–0.102^{+} (0.057)
9 to 12	0.016 (0.049)
12 to 15	0.027 (0.065)
15 to 18	ref. cat.
18 to 21	0.044 (0.065)
21 to 24	0.019 (0.065)
24 to 27	0.198** (0.068)
over 27	0.286** (0.093)

The coefficients show the impact of early pregnancy temperature exposure by temperature category. The coefficients represent the effect of one additional day with a given mean temperature on the clinically unobserved pregnancy loss rate relative to a day with a mean temperature of 15–18°C. The early pregnancy period is defined as a six-week-long period starting after the week of conception. The outcome variable is the clinically observed conception rate per week per 100,000 women aged 16–44 years, which is calculated using conceptions that end in clinically observed pregnancy outcomes. The impacts of temperature exposure on the clinically unobserved pregnancy loss rate are obtained by multiplying the estimated temperature coefficients by −1. The model has county-by-year fixed effects, region-by-calendar-week fixed effects, and region-by-calendar-week-specific quadratic time trends. Precipitation, pre-conception weather, and the share of non-working days are controlled for. We weight by the counties' average female population size (aged 16–44 years) between 1981 and 2015. Standard errors are shown in parenthesis, clustered by county and time. $^{+}p<0.10$, $^{*}p<0.05$, $^{**}p<0.01$

Supplementary Table S5: Placebo regressions with weather 1 or 2 years later of the actual exposure period

	(1)	(2)
Daily mean temperature (°C)	Weather 1 year later	Weather 2 years later
below –5	0.072 (0.074)	–0.107 (0.085)
–5 to 0	–0.188* (0.067)	–0.012 (0.065)
0 to 5	–0.020 (0.053)	–0.027 (0.058)
5 to 10	–0.008 (0.054)	–0.117* (0.053)
10 to 15	0.052 (0.038)	–0.011 (0.039)
15 to 20	ref. cat.	ref. cat.
20 to 25	0.011 (0.034)	–0.009 (0.037)
over 25	–0.005 (0.049)	–0.043 (0.048)

The coefficients show the impact of early pregnancy temperature exposure by temperature category using weather variables measured exactly 1 or 2 years later of the actual exposure period. The coefficients represent the effect of one additional day with a given mean temperature on the clinically unobserved pregnancy loss rate relative to a day with a mean temperature of 15–20°C. The early pregnancy period is defined as a six-week-long period starting after the week of conception. The outcome variable is the clinically observed conception rate per week per 100,000 women aged 16–44 years, which is calculated using conceptions that end in clinically observed pregnancy outcomes. The impacts of temperature exposure on the clinically unobserved pregnancy loss rate are obtained by multiplying the estimated temperature coefficients by −1. The model has county-by-year fixed effects, region-by-calendar-week fixed effects, and region-by-calendar-week-specific quadratic time trends. Precipitation, pre-conception weather, and the share of non-working days are controlled for. We weight by the counties' average female population size (aged 16–44 years) between 1981 and 2015. Standard errors are shown in parenthesis, clustered by county and time. $^{+}p<0.10$, $^{*}p<0.05$, $^{**}p<0.01$

Supplementary Table S6: Impact of early pregnancy temperature exposure on the clinically unobserved pregnancy loss rate: excluding week 1

Daily mean temperature (°C) (week 2-6)	(1)
below –5	0.002 (0.088)
–5 to 0	–0.215** (0.072)
0 to 5	–0.090 (0.066)
5 to 10	–0.138* (0.050)
10 to 15	0.026 (0.040)
15 to 20	ref. cat.
20 to 25	0.030 (0.047)
over 25	0.202** (0.052)

The coefficients show the impact of early pregnancy temperature exposure by temperature category (excluding week 1 after the conception week). The coefficients represent the effect of one additional day with a given mean temperature on the clinically unobserved pregnancy loss rate relative to a day with a mean temperature of 15–20°C. The early pregnancy period is defined as a five-week-long period starting with the second week after the week of conception. The estimations come from equation (12). The outcome variable is the clinically observed conception rate per week per 100,000 women aged 16–44 years, which is calculated using conceptions that end in clinically observed pregnancy outcomes. The impacts of temperature exposure on the clinically unobserved pregnancy loss rate are obtained by multiplying the estimated temperature coefficients by −1. The model has county-by-year fixed effects, region-by-calendar-week fixed effects, and region-by-calendar-week-specific quadratic time trends. Precipitation, pre-conception weather, weather in the first week of the pregnancy, and the share of non-working days are controlled for. We weight by the counties' average female population size (aged 16–44 years) between 1981 and 2015. Standard errors are shown in parenthesis, clustered by county and time.
$^{+}p<0.10$, $^{*}p<0.05$, $^{**}p<0.01$

Supplementary Table S7: Impact of early pregnancy temperature exposure on the conception rate calculated from pregnancies ending in clinically observed spontaneous foetal losses

Daily mean temperature (°C)	(1)
below –5	–0.014 (0.012)
–5 to 0	0.016 (0.010)
0 to 5	–0.006 (0.008)
5 to 10	–0.002 (0.009)
10 to 15	0.004 (0.008)
15 to 20	ref. cat.
20 to 25	0.004 (0.007)
over 25	–0.023* (0.011)

The coefficients show the impact of early pregnancy temperature exposure by temperature category. The coefficients represent the effect of one additional day with a given mean temperature on the conception rate calculated from pregnancies ending in clinically observed spontaneous foetal losses relative to a day with a mean temperature of 15–20°C. The early pregnancy period is defined as a six-week-long period starting after the week of conception. The outcome variable is the conception rate calculated from pregnancies ending in clinically observed spontaneous foetal losses per week per 100,000 women aged 16–44 years. The model has county-by-year fixed effects, region-by-calendar-week fixed effects, and region-by-calendar-week-specific quadratic time trends. Precipitation, pre-conception weather, and the share of non-working days are controlled for. We weight by the counties' average female population size (aged 16–44 years) between 1981 and 2015. Standard errors are shown in parenthesis, clustered by county and time. $^{+}p<0.10$, $^{*}p<0.05$, $^{**}p<0.01$
